# First-line Advanced Cutaneous Melanoma Treatments: Where Do We Stand?

**DOI:** 10.2196/29912

**Published:** 2021-12-15

**Authors:** Louay S Abdulkarim, Richard J Motley

**Affiliations:** 1 Department of Dermatology Cardiff University Cardiff United Kingdom; 2 Welsh Institute of Dermatology Cardiff United Kingdom

**Keywords:** advanced cutaneous melanoma, first-line treatments, immunotherapy, targeted therapy, combinational therapy, dermatologic adverse events, cutaneous side effects

## Abstract

Cutaneous melanoma has always been a dreaded diagnosis because of its high mortality rate and its proclivity for invasiveness and metastasis. Historically, advanced melanoma treatment has been limited to chemotherapy and nonspecific immunotherapy agents that display poor curative potential and high toxicity. However, during the last decade, the evolving understanding of the mutational burden of melanoma and immune system evasion mechanisms has led to the development of targeted therapy and specific immunotherapy agents that have transformed the landscape of advanced melanoma treatment. Despite the considerable strides in understanding the clinical implications of these agents, there is a scarcity of randomized clinical trials that directly compare the efficacy of the aforementioned agents; hence, there are no clear preferences among the available first-line options. In addition, the introduction of these agents was associated with a variety of dermatologic adverse events, some of which have shown a detrimental effect on the continuity of treatment. This holds especially true in light of the current fragmentation of care provided by the managing health care professionals. In this study, we attempt to summarize the current understanding of first-line treatments. In addition, the paper describes the indirect comparative evidence that aids in bridging the gap in the literature. Furthermore, this paper sheds light on the impact of the scarcity of dermatology specialist input in the management of dermatologic adverse events associated with advanced melanoma treatment. It also looks into the potential avenues where dermatologic input can bridge the gap in the care provided by oncologists, thus standardizing the care provided to patients with melanoma presenting with dermatologic adverse events.

## Introduction

Melanoma is a malignant transformation of the melanocytes. It accounts for approximately 1% of all skin cancers; however, it carries the highest mortality rate among all skin cancers [1,2]. The high mortality rate of melanoma is mainly because of its early metastatic potential and aggressive nature [3]. Surgery has been shown to be a successful treatment for localized melanomas; however, advanced cases have a grim prognosis [3]. In the last decade, medical management of advanced melanoma has transformed the life expectancy of patients with melanoma. The introduction of novel agents, namely immunotherapy and targeted therapy, has increased the median overall survival (OS) by 10-fold, from an average of 6 months to >5 years [4,5]. Targeted therapy comprises agents that directly inhibit mutated kinases, namely BRAF and mitogen-activated protein kinase kinase, which have been implicated in the growth and survival of cancerous melanocytes. However, the efficacy of BRAF inhibitor (BRAFi) and mitogen-activated protein kinase kinase inhibitor (MEKi) monotherapies is limited by early resistance and an upsurge in treatment-associated skin tumors. Consequently, a combined BRAFi plus MEKi approach was trialed, which resulted in superior survival rates while minimizing the aforementioned limitations.

In addition, specific immunotherapy agents were developed following Nobel Prize-winning discoveries that outlined the pivotal role of certain immune downregulatory signals that facilitate tumor growth. Hitherto, several single and combined treatments have been approved as first-line therapy for advanced melanoma.

It is worth mentioning that BRAF status testing is imperative to the treatment choice; in general, immunotherapy is offered to both patients with BRAF-positive and BRAF-negative melanoma, whereas targeted therapy (BRAFi and MEKi) is only used for patients who test positive for the BRAF mutation [6-8].

## Immunotherapy in Clinical Practice

Currently, there are 3 types of immunotherapy treatments approved for unresectable or metastatic melanoma treatment regardless of the BRAF status: 2 anti–programmed death 1 (PD-1) agents, namely nivolumab and pembrolizumab; a single anti–programmed death 1 ligand (PD-L1) agent, atezolizumab; and a single anti–cytotoxic T-lymphocyte-associated protein 4 (CTLA4) agent, ipilimumab [6,7,9].

CheckMate 067, a phase 3 double-blind randomized controlled trial (RCT), demonstrated the superiority of nivolumab with or without ipilimumab over ipilimumab monotherapy. Because of the study design, nivolumab plus ipilimumab combination therapy was not directly tested against nivolumab monotherapy. However, indirect analysis suggested that adding ipilimumab to nivolumab monotherapy achieved higher progression-free survival (PFS) and response rates, whereas no significant difference was reported in OS (Table 1) [5]. Therefore, both nivolumab-containing groups have been approved as first-line treatments [6,7].

**Table 1:** Summary of the 5-year efficacy results of CheckMate 067 along with the reported dermatologic adverse events^a^.

Study group		Nivolumab plus ipilimumab	Nivolumab	Ipilimumab
**Overall survival**				
	Value, median (months)	>60	36.9	19.9
	HR^b^	0.52^c^	0.63^d^	N/A^e^
**Progression-free survival**				
	Value, median (months)	11.5	6.9	2.9
	HR	0.42^c^	0.53^d^	N/A
Adverse events (all grade), %		96	87	86
Adverse events (grade ≥3), %		59	23	28
**Dermatologic adverse events**				
	Rash (all grade), %	30	24	22
	Rash (grade ≥3), %	3	<1	2
	Pruritus (all grade), %	36	23	36
	Pruritus (grade ≥3), %	2	<1	<1
	Vitiligo (all grade), %	9	11	5
	Vitiligo (grade ≥3), %	0	<1	0
	Dry skin (all grade), %	5	5	4
	Dry skin (grade ≥3), %	0	0	0
	Maculopapular rash (all grade), %	12	5	12
	Maculopapular rash (grade ≥3), %	2	1	<1

^a^Adapted from Larkin et al [5].

^b^HR: hazard ratio.

^c^Nivolumab plus ipilimumab versus ipilimumab.

^d^Nivolumab versus ipilimumab.

^e^N/A: not applicable.

However, the enhanced efficacy of combined immunotherapy comes with added adverse events [5]. Therefore, the choice between combined and single agent immunotherapy must be tailored to the patient’s circumstances, considering different factors, such as the patient’s health status (absence of autoimmune diseases or other comorbidities that might aggravate the immune-related adverse events) and the patient’s willingness to tolerate the added toxicity associated with combination therapy. Furthermore, the availability of support services that can monitor and manage adverse events should be considered [7].

Patients with advanced melanoma were recruited in CheckMate 067 regardless of the tumor’s BRAF status; hence, nivolumab plus ipilimumab combination therapy and nivolumab monotherapy were approved for both BRAF-positive and BRAF-negative melanomas. Of note, the percentage of BRAF-positive melanomas in CheckMate 067 was 31.5%, which is lower than the reported prevalence of BRAF mutations among patients with melanoma (approximately 60%) [5,10]. Hence, the overall results might be a misrepresentation of the BRAF-positive subgroup which are known to have worse prognosis.

In KEYNOTE-006, a phase 3 open label RCT, pembrolizumab monotherapy has been shown to improve PFS, OS, and response rates compared with ipilimumab monotherapy (Table 2) [11]. As with nivolumab monotherapy, pembrolizumab monotherapy is recommended as a first-line therapy if the added side effects of combination immunotherapy cannot be tolerated [6,7]. The tolerable adverse events profile of pembrolizumab paralleled with its associated long-term survival rate nominates it as a potential candidate for combined immunotherapy and combined targeted therapy plus immunotherapy. However, there are no published data that support its use in a combined regimen.

**Table 2:** Summary of the 5-year efficacy results of KEYNOTE-006 along with the reported dermatologic adverse events^a^.

Study group			Pembrolizumab^b^		Ipilimumab
**Overall survival**					
	Value, median (months)	32.7		15.9	
	HR^c^	0.75^d^		N/A^e^	
**Progression-free survival**					
	Value, median (months)	8.4		3.4	
	HR	0.57^d^		N/A	
Adverse events (all grade), %			77-82		74
Adverse events (grade ≥3), %			17		20
**Dermatologic adverse events**					
	Rash (all grade), %	16-17		16	
	Rash (grade ≥3), %	0		0	
	Pruritus (all grade), %	20		26	
	Pruritus (grade ≥3), %	0		0	

^a^Adapted from Schachter et al [12] and Robert et al [11].

^b^Compiled results of the 2 pembrolizumab doses studied in KEYNOTE-006.

^c^HR: hazard ratio.

^d^Pembrolizumab versus ipilimumab.

^e^N/A: not applicable.

To date, the following are approved first-line immunotherapy treatments for unresectable or metastatic melanoma irrespective of BRAF mutation status: nivolumab plus ipilimumab combination, nivolumab monotherapy, and pembrolizumab monotherapy [8]. Patients with BRAF-positive advanced melanoma are offered additional first-line treatment options, namely combined BRAFi plus MEKi regimens, as discussed below.

## Targeted Therapy in Clinical Practice

In total, 3 BRAFi have been approved for unresectable or metastatic melanoma, namely vemurafenib, dabrafenib, and encorafenib. In addition, 3 MEKi, namely cobimetinib, trametinib, and binimetinib, have been approved for use along with the aforementioned BRAFi agents. The superiority of combined BRAFi plus MEKi therapy over BRAFi monotherapy was established in the coBRIM, COMBI-d, COMBI-v, and COLUMBUS RCTs (Tables 3-5) [13-15]. Moreover, the addition of MEKi to BRAFi monotherapies has been shown to mitigate the high resistance rates and high toxicities associated with BRAFi monotherapy and overcome the limited response rates and early resistance in MEKi monotherapies. In light of these results, BRAFi plus MEKi combination supplanted targeted monotherapy regimens as first-line systemic treatments for advanced melanoma [16-19]. To date, there is no evidence available from head-to-head trials that compare the 3 approved BRAFi plus MEKi combination regimens, namely vemurafenib plus cobimetinib, dabrafenib plus trametinib, and encorafenib plus binimetinib. The following section attempts to compare these lines of treatment using indirect and comparative analyses.

**Table 3:** Summary of the coBRIM efficacy results along with the reported dermatologic adverse events^a^.

Study group			Cobimetinib plus vemurafenib		Vemurafenib
**Overall survival**					
	Value, median (months)	22.3		17.4	
	HR^b^	0.70^c^		N/A^d^	
**Progression-free survival**					
	Value, median (months)	12.3		7.2	
	HR	0.58^c^		N/A	
Adverse events (all grade), %			99.2		98
Adverse events (grade ≥3), %			75.3		61.4
**Dermatologic adverse events**					
	Rash (all grade), %	72.5		67.5	
	Rash (grade ≥3), %	17		16.3	
	Photosensitivity (all grade), %	47.8		37.8	
	Photosensitivity (grade ≥3), %	4.5		0	
	Alopecia (all grade), %	16.6		30.5	
	Alopecia (grade ≥3), %	0.4		0.4	
	Hyperkeratosis (all grade), %	10.1		27.2	
	Hyperkeratosis (grade ≥3), %	0.4		2.4	
	Squamous cell carcinoma (all grade), %	4		12.6	
	Squamous cell carcinoma (grade ≥3), %	3.6		12.6	
	Keratoacanthoma (all grade), %	1.6		9.3	
	Keratoacanthoma (grade ≥3), %	1.2		8.5	

^a^Adapted from Ascierto et al [13].

^b^HR: hazard ratio.

^c^Cobimetinib plus vemurafenib versus vemurafenib.

^d^N/A: not applicable.

**Table 4:** Summary of the COMBI-d efficacy results along with the reported dermatologic adverse events^a^.

Study group		Dabrafenib plus trametinib	Dabrafenib
**Overall survival**			
	Value, median (months)	25.1	18.7
	HR^b^	0.71^c^	N/A^d^
**Progression-free survival**			
	Value, median (months)	11.0	8.8
	HR	0.67^c^	N/A
Adverse events (all grade), %		87	90
Adverse events (grade ≥3), %		32	30
**Dermatologic adverse events**			
	Rash (all grade), %	24	20
	Rash (grade ≥3), %	0	<1
	Dry skin (all grade), %	9	14
	Dry skin (grade ≥3), %	0	0
	Pruritus (all grade), %	7	11
	Pruritus (grade ≥3), %	0	0
	Alopecia (all grade), %	5	26
	Alopecia (grade ≥3), %	0	0
	Hyperkeratosis (all grade), %	6	33
	Hyperkeratosis (grade ≥3), %	0	<1
	Skin papilloma (all grade), %	1	18
	Skin papilloma (grade ≥3), %	0	0
	Dermatitis acneiform (all grade), %	8	3
	Dermatitis acneiform (grade ≥3), %	0	0
	Squamous cell carcinoma (all grade), %	3	9
	Squamous cell carcinoma (grade ≥3), %	3	9
	New primary melanoma (all grade), %	<1	2
	New primary melanoma (grade ≥3), %	<1	<1

^a^Adapted from Long et al [14].

^b^HR: hazard ratio.

^c^Dabrafenib plus trametinib versus trametinib.

^d^N/A: not applicable.

**Table 5:** Summary of the COLUMBUS efficacy results along with the reported dermatologic adverse events^a^.

Study group			Encorafenib plus binimetinib		Encorafenib		Vemurafenib
**Overall survival**							
	Value, median (months)	33.6		23.5		16.9	
	HR^b^	0.61^c^		0.76^d^		N/A^e^	
**Progression-free survival**							
	Value, median (months)	14.9		9.6		7.3	
	HR	0.51^c^		0.68^d^		N/A	
Adverse events (all grade), %			98.4		99.5		100
Adverse events (grade ≥3), %			68.2		67.7		65.6
**Dermatologic adverse events**							
	Rash (all grade), %	16.1		20.8		30.1	
	Rash (grade ≥3), %	1.6		2.1		3.2	
	Pruritus (all grade), %	12.5		21.9		10.8	
	Pruritus (grade ≥3), %	0.5		0.5		0	
	Hyperkeratosis (all grade), %	15.1		40.1		29	
	Hyperkeratosis (grade ≥3), %	0.5		3.6		0	
	Dry skin (all grade), %	16.1		30.2		23.1	
	Dry skin (grade ≥3), %	0		0.5		0	
	Alopecia (all grade), %	14.6		56.3		37.6	
	Alopecia (grade ≥3), %	0		0		0	
	Palmoplantar erythrodysesthesia syndrome (all grade), %	7.3		51.6		14	
	Palmoplantar erythrodysesthesia syndrome (grade ≥3), %	0		13.5		1.1	
	Photosensitivity (all grade), %	3.6		3.6		25.3	
	Photosensitivity (grade ≥3), %	0.5		0		1.1	
	Palmoplantar keratoderma (all grade), %	9.9		26.6		17.7	
	Palmoplantar keratoderma (grade ≥3), %	0		2.1		1.1	
	Keratosis pilaris (all grade), %	4.7		3.6		25.3	
	Keratosis pilaris (grade ≥3), %	0.5		0		1.1	
	Papilloma^f^ (all grade), %	7		10		19	
	Papilloma^f^ (grade ≥3), %	N/A		N/A		N/A	
	Squamous cell carcinoma^f^ (all grade), %	3		8		17	
	Squamous cell carcinoma^f^ (grade ≥3), %	N/A		N/A		N/A	
	Basal cell carcinoma^f^ (all grade), %	2		1		2	
	Basal cell carcinoma^f^ (grade ≥3), %	N/A		N/A		N/A	

^a^Adapted from Ascierto et al [15] and Gogas et al [20].

^b^HR: hazard ratio.

^c^Encorafenib plus binimetinib versus vemurafenib.

^d^Encorafenib versus vemurafenib.

^e^N/A: not applicable.

^f^These dermatologic adverse events were reported separately by Gogas et al [20] as all grade dermatologic adverse events with no further breakdown.

## Comparing Current Targeted Therapy Combinations

To date, no direct studies have been conducted that would prioritize dabrafenib plus trametinib over vemurafenib plus cobimetinib or vice versa. coBRIM, which compared vemurafenib plus cobimetinib and vemurafenib monotherapy, and COMBI-v, which compared dabrafenib plus trametinib and vemurafenib monotherapy, share some similarities in study design features and control groups. On the basis of these similarities, Galván‐Banqueri et al [21] conducted an indirect comparison between the 2 combined regimens and concluded that there were no significant differences in OS and PFS. The similarities in PFS and OS were also reported in a systematic review and network meta-analysis by Garzón‐Orjuela et al [22]. However, this study highlighted disparities in safety profiles; dabrafenib plus trametinib was found to be safer because of the lower risk of grade 3 and grade 4 adverse events, such as ocular adverse events (serous retinopathy) and elevated liver enzymes.

Indirect comparisons should be interpreted cautiously, as even similarly designed trials might exhibit some degree of discrepancy that would discredit any conclusions made. In case of coBRIM and COMBI-v, there were differences in the inclusion criteria, study end points (PFS was the primary end point in coBRIM and secondary in COMBI-d), and allowance of patient crossover between study arms [13,23].

In COLUMBUS, a phase 3 open label RCT, encorafenib plus binimetinib displayed unprecedented efficacy rates for a BRAFi plus MEKi combination therapy (median OS of 33.6 months and median PFS of 14.9 months), especially in median OS. In comparison, dabrafenib plus trametinib treatment achieved a median OS of 25.1 months and a median PFS of 11 months, which was similar to the vemurafenib plus cobimetinib combination results, yielding 22.3 and 12.3 months for median OS and PFS, respectively (Tables 3-5) [13-15].

The National Institute for Health and Care Excellence (UK) recruited Pierre Fabre, a pharmaceutical company, to compare the clinical efficacy and cost-effectiveness of encorafenib plus binimetinib and dabrafenib plus trametinib by evaluating the direct and indirect evidence. The results showed that there were no significant differences in the clinical outcomes between the 2 BRAFi plus MEKi combinations; however, encorafenib plus binimetinib was shown to be more cost-effective. Hence, it was recommended by the National Institute for Health and Care Excellence for BRAF-positive advanced melanomas [24].

The study designs of COLUMBUS, coBRIM, COMBI-d, and COMBI-v had a notable difference in patient characteristics, which might suggest the added benefit of certain targeted therapy combinations in select patient subcategories. Unlike coBRIM, COMBI-d, and COMBI-v, the COLUMBUS trial allowed the recruitment of previously treated patients, including those who were previously treated with BRAFi monotherapies [13,25-27]. This shows that the clinical outcomes were achieved in a cohort that might have developed resistance or progressed with previous BRAFi agents. It also enhances the external validity of the results and establishes encorafenib plus binimetinib as an effective second-line treatment for patients who have progressed in previous systemic treatments.

Of note, the number of patients with elevated levels of lactate dehydrogenase (a negative prognostic factor) involved in COLUMBUS was lower than in other trials, which might indicate that the patients enrolled had a *healthier* baseline. However, apart from the disparity in lactate dehydrogenase levels, the other prognostic factors were comparable. In addition, vemurafenib monotherapy was a common control group in COLUMBUS, COMBI-v, and coBRIM and produced comparable results, which negates any significant differences between study participants [13,25,26].

Pharmacokinetic analysis of the available BRAFi revealed significant differences. Delord et al [28] compared encorafenib, dabrafenib, and vemurafenib in a preclinical setting (cell lines and xenograft melanoma tumors) and showed that although all 3 agents were able to inhibit BRAF V600E kinase activity at the same concentration, encorafenib had a markedly prolonged half-life (>30 hours) compared with that of dabrafenib (2 hours) and vemurafenib (0.5 hours). This translated to increased drug availability, prolonged target suppression, and enhanced potency. Delord et al [28] demonstrated the increased potency of encorafenib by showing that the half-maximal inhibitory concentration (IC_50_) was achieved with a lower concentration of encorafenib (<40 nmol/L) compared with that of dabrafenib (<100 nmol/L) and vemurafenib (<1 μmol/L) [28]. The prolonged half-life and superior potency of encorafenib might explain the prolonged median OS of encorafenib plus binimetinib evident in the COLUMBUS trial. Additional research should delineate the impact of the pharmacokinetic profile on the onset and overall onset of resistance, a notable limiting factor of BRAFi and MEKi [17].

The frequency of certain dermatologic adverse events varied considerably between the monotherapy groups in the COLUMBUS trial and across other BRAF trials, which might point to the presence of molecular differences in same-group agents (Tables 3-5) [13-15]. One such difference is the variability of kinase inhibition among BRAF isotypes. Encorafenib was shown to exhibit similar inhibition on both mutated and wild-type BRAF isotypes, whereas both dabrafenib and vemurafenib inhibited mutated BRAF kinase more efficiently with minimal inhibition of wild-type BRAF kinase [29]. The uneven inhibition leads to the hyperstimulation of wild-type BRAF kinase manifesting clinically as the paradoxical rise of BRAFi-associated dermatologic adverse events, such as squamous cell carcinoma, primary melanoma, and papillomas [29,30]. Adelmann et al [29] introduced the term *paradox indices*, which estimates a therapeutic window that represents the concentration range within which maximum inhibition of BRAF is achieved while maintaining the lowest paradoxical activation of the downstream kinase extracellular signal-regulated kinase (ERK), the culprit kinase that drives treatment-induced dermatologic adverse events in wild-type BRAF tissues [31]. Encorafenib had the highest paradox index (50), representing the most potent agent with the widest safety margin, followed by those of dabrafenib (10) and vemurafenib (5.5) [29]. The clinical results corresponded with the reported paradox indices, as vemurafenib-associated squamous cell carcinoma was twice as common compared with the encorafenib group; similar disparities were noted in papilloma and keratosis pilaris (Table 5).

The unique pharmacokinetic profile of encorafenib could also explain the disparity in the prevalence of nondermatologic adverse events. For instance, pyrexia was shown to be the most common adverse event and a substantial limiting factor among patients treated with dabrafenib plus trametinib, causing the most treatment interruptions (30%), dose reductions (14%), and permanent terminations (3%) [26]. COLUMBUS trial revealed a sizable decrease in pyrexia incidence in the encorafenib plus binimetinib group (18%) compared with that in the dabrafenib plus trametinib group (53%) in the COMBI-v trial [20,26]. In addition, COLUMBUS showed that vemurafenib (an agent used in the vemurafenib plus cobimetinib combination) monotherapy group had an approximately 2-fold increase in pyrexia (30%) compared with the encorafenib monotherapy group (16%) [20]. Both findings suggest that encorafenib plus binimetinib is, potentially, the safest BRAFi plus MEKi currently offered for treatment-induced pyrexia. Given the lack of direct evidence, detailed comparisons of other critical adverse events, especially those that impose the greatest threat of treatment interruption, are much needed to help navigate the available treatments. To date, all 3 combinations have been approved as first-line treatments for BRAF-positive advanced melanoma, especially in rapidly deteriorating cases [6,32].

## Immunotherapy Versus Targeted Therapy

To date, no evidence is available from head-to-head trials that compare immunotherapy and targeted therapy for BRAF-positive melanomas. Ugurel et al [33] conducted an exploratory analysis comparing the PFS and OS of landmark trials assessing advanced melanoma treatments. The study included 25 prospective clinical trials from 2002 to 2017, producing 83 Kaplan-Meier survival curves. Ugurel et al [33] showed that there was a high concordance among the survival curves of different agents within the same group of both targeted and immunotherapy agents used as first-line therapies. However, the survival data of the second or higher treatment lines showed lower concordance. Moreover, the combined BRAFi plus MEKi had superior PFS rates compared with those of combined immunotherapy at 6 months (72.3% vs 63.8%). In addition, the OS rates of combined BRAFi plus MEKi were also higher at 12 months (76.6%) than those of the combined immunotherapy (73.1%). However, the OS rate curves crossed over in favor of combined immunotherapy at 24 months, yielding 62.9% compared with 53.3% in combined BRAFi plus MEKi [33]. It is worth mentioning that the analysis of Ugurel et al [33] only included trials that evaluated treatments of BRAF-positive melanoma that were published up to January 1, 2017; hence, the results of the aforementioned analysis did not account for agents approved more recently, such as encorafenib plus binimetninb.

Moreover, the 5-year update of CheckMate 067 demonstrated the long-term survival benefit of nivolumab groups in patients with BRAF-positive melanoma. The combination arm reported a median OS of >60 months (median OS has not been reached yet), representing the longest median OS of all the currently available first-line treatments, followed by nivolumab monotherapy, which achieved a median OS of 45.5 months (Tables 1-5) [5]. Conversely, the 5-year combined pooled data of COMBI-d and COMBI-v revealed that the median OS at 5 years was 25.9 months in patients with BRAF-positive melanoma on combined dabrafenib plus trametinib treatment [23]. Comparing the results from Checkmate 067 and COMBI-v or COMBI-d would not present tangible evidence because of the discrepancy in the characteristics of study populations [5,23].

The inferior 24-month survival outcome of targeted therapy reported in the analysis of Ugurel et al [33] and the considerable difference in the 5-year median survival between the nivolumab groups and the dabrafenib plus trametinib combination group delineate the acquired resistance phenomenon associated with targeted therapy, which became eminent approximately 6 months after treatment initiation [5,17,23].

Similarly, the lower PFS and OS rates of immunotherapy during the first year of treatment depicted in the findings of Ugurel et al [33] displayed the primary resistance phenomenon associated with immunotherapy agents [34]. It is worth mentioning that the 5-year compiled data of CheckMate 067 denote a steadily increasing rate in complete response, regardless of the BRAF status, which might suggest the reversibility of immunotherapy-associated resistance [5].

Furthermore, studies have shown that BRAFi plus MEKi agents displayed a more pronounced therapeutic effect in patients with high lactate dehydrogenase. Conversely, immunotherapy was more effective in patients with normal levels of lactate dehydrogenase [35,36].

These findings suggest the superiority of combined BRAFi plus MEKi as an *acute* treatment especially in aggressive melanomas, while supporting the superior role of immunotherapy as a *maintenance* therapy. Furthermore, these findings suggest the benefit of sequential therapy, where treatment could be initiated by BRAFi plus MEKi and then maintained by immunotherapy, thus harvesting the benefits of both lines of therapy. This approach is corroborated by the 5-year analysis of the pooled data of COMBI-d and COMBI-v trials, which showed that a complete response was observed in patients who were treated with immunotherapy following dabrafenib plus trametinib therapy administered in the aforementioned trials [23]. This regimen is currently being studied in ImmunoCobiVem (ClinicalTrials.gov NCT02902029), a clinical trial assessing the efficacy and safety of sequential treatment with cobimetinib plus vemurafenib followed by atezolizumab.

The European Society for Medical Oncology recommends the use of immunotherapy in unresectable melanoma regardless of the BRAF mutation status, as long as the immunotherapy can be safely administered, meaning that melanoma is not progressing very quickly and there is no imminent threat to any function or organ [6]. The US National Comprehensive Cancer Network recommends both immunotherapy and targeted therapy as first-line treatments for unresectable melanoma; however, targeted therapy is preferred for rapidly deteriorating BRAF-positive melanomas [7]. Studies comparing targeted and immunotherapy agents as first-line treatment are yet to be published; such results will conceivably shape the guidelines of this dynamic field.

## Combined Targeted and Immunotherapy Regimen

On July 30, 2020, the United States Food and Drug Administration approved atezolizumab combined with vemurafenib plus cobimetinib as first-line treatment for unresectable melanoma. This is the first approved combined treatment regimen that incorporates targeted therapy and immunotherapy [9].

Atezolizumab is a PD-L1 inhibitor that has been approved as a monotherapy to treat other solid cancers, including breast and urothelial cancers [37,38]. Atezolizumab monotherapy has also been investigated in a phase 1 trial for the treatment of advanced melanoma. In this study, Hamid et al [39] showed that atezolizumab achieved a median OS of 23 months. In addition, the median response duration exceeded 5 years, while maintaining a tolerable safety profile. The response durability and tolerability presented atezolizumab as a promising agent for melanoma treatment.

However, the recent approval of atezolizumab was based on the results of IMspire150, a phase 3 double-blind RCT that assessed the efficacy and safety of atezolizumab plus vemurafenib plus cobimetinib versus vemurafenib plus cobimetinib plus placebo. Both arms were initially treated with the vemurafenib plus cobimetinib combination for the first cycle (a 28-day cycle), after which the intervention group was commenced on atezolizumab, whereas the control group was given a matched placebo. The PFS of the triple agent group was 15.1 months, which was significantly longer than that of the dual agent group (10.6 months) [40]. Interestingly, the PFS curves of the 2 groups parted ways after 7 months of treatment, at approximately the same time that the acquired resistance of BRAFi plus MEKi becomes apparent, highlighting the added benefit of incorporating immunotherapy with combined targeted therapy [17,33,40]. In addition, the median duration of response was prolonged in the triple agent arm. At the time of the interim analysis, the death rate of the triple agent group was 36% compared with 43% in the control group. Accordingly, the OS rate at 24 months was predicted to be 60% and 53% for the triple and dual agent groups, respectively [40].

Notably, immune-mediated adverse events, which required systemic corticosteroids, were more frequent in the triplet group. These adverse events include dermatitis acneiform, acne, pneumonitis, uveitis, hyperthyroidism, and raised liver enzymes. Other dermatologic adverse events, such as photosensitivity reactions, rash, pruritus, dry skin, and sunburn were also reported in the triplet group [40].

The higher toxicity of the triple agent treatment was also portrayed in KEYNOTE-022, a phase 2 double-blind RCT that evaluated the addition of pembrolizumab to dabrafenib plus trametinib combined therapy. The study showed that the triple agent group had a superior median PFS of 16.0 months versus 10.3 months in the dabrafenib plus trametinib only group. However, the *P* value threshold for statistical significance (*P*=.003) was not achieved for PFS (*P*=.04). However, the triple agent group had a higher rate of patients with complete response (18.3%) compared with that in the dual agent group (13.3%). Furthermore, the triple agent group displayed improved response duration; however, it was associated with higher toxicity, leading to more frequent treatment discontinuations [41].

In light of the added toxicity of the triple agent approach and the lack of mature data that demonstrate the OS benefit of the triple therapy, guidelines are yet to outline the exact role of this regimen in treating BRAF-positive melanoma and the implications it has on the currently available dual agent options [42]. The results of other ongoing trials that evaluate the triple agent approach, such as ImmunoCobiVem (ClinicalTrials.gov NCT02902029) and COMBI-I (ClinicalTrials.gov NCT02967692), will aid in delineating the role of combined immunotherapy and targeted therapy in melanoma treatment.

Treatment-associated toxicity is pivotal in shaping current and future guidelines, particularly for adverse events that have been detrimental to treatment continuation. In fact, treatment-associated dermatologic adverse events have been ranked high for both frequency and severity. Dermatologic adverse events present in approximately 50% of patients with advanced melanoma treated with immunotherapy. Targeted therapy-related dermatologic adverse events occur in 90% of the patients who are treated, rendering dermatologic adverse events not only one of the most frequently reported adverse events but also one of the most common reasons for treatment interruption [32,43]. Immunotherapy-related dermatologic adverse events include maculopapular rash, vitiligo, and pruritus [44]. In contrast, dermatologic adverse events associated with targeted therapy are not only more common but also are more clinically relevant [32]. Targeted therapy-induced dermatologic adverse events, which are responsible for most treatment interruptions include proliferative cutaneous neoplasms, rash, and photosensitivity reactions [45].

## Dermatologic Adverse Events: A Challenge in Clinical Practice

Advanced melanoma treatment-related dermatologic adverse events are mainly managed by oncologists and dermatologists. However, the former are more involved in the management of dermatologic adverse events, as advanced cutaneous melanoma cases are referred to oncology care, with minimal care provided by dermatologists.

Furthermore, the literature shows that there is hesitancy in requesting dermatology input when managing dermatologic adverse events despite the challenges that they present in clinical practice, including dose reduction or, more importantly, treatment interruption or termination [46-48]. The following are 3 studies that showcase this phenomenon and illustrate the degree of dermatology specialist input in managing oncology treatment-related dermatologic adverse events.

In a French study by Peuvrel et al [46], 67 nondermatologist health care professionals who manage patients with cancer on targeted therapy were surveyed. Although there was consistency in treating common, uncomplicated cases, greater disparity was evident in managing complex cases, such as secondary skin infection or cases associated with radiodermatitis. Moreover, the study revealed that dermatologic consultations were prompted mainly if dermatologic adverse events were exacerbated or were persistent for >2 weeks. It also identified that nondermatologists struggled to grade dermatologic adverse events and manage those located in skin appendages, such as nails and the scalp. Less than half of the respondents would refer to a dermatologist if they needed help in managing cutaneous side effects.

The disparity in management and latency in seeking specialist input was echoed in a German study by Hassel et al [47] where oncologists and dermato-oncologists were provided with pictures and medical history of a patient with an acneiform rash, a dermatologic adverse event associated with targeted therapy and were asked to provide information on grading and treatment strategies. The results showed that dermato-oncologists had a more liberal use of local antibiotics (*P*=.006) and isotretinoin (*P*=.002). However, the data showed that dermato-oncologists delayed targeted therapy less often because of skin toxicity (*P*=.009). Despite these discrepancies, only 9% of the oncologists referred the patient to a dermatologist [47].

Finally, in the Unites States, Boone et al [48] surveyed 110 oncology clinicians who manage patients on targeted therapy. Of the health practitioners surveyed, 17% reported rash in approximately 90% of their patients: 32% had terminated treatment because of rash, and 60% had to reduce the dose. Despite the high rate of rash causing considerable treatment disruptions, only 8% of those surveyed requested dermatology consultations and fewer than half actively treated mild rashes [48].

Although it might be inappropriate to draw generalizations from questionnaire-based studies, the aforementioned studies provide insight into the oncology practice in different parts of the world. All of these studies revealed a delay in seeking dermatology consultations despite facing challenging dermatologic adverse events that led to treatment disruptions. However, the questionnaires did not account for the impact of late dermatology consultations on the physical and psychological well-being of the patients, nor did they account for the implications of any untreated dermatologic adverse events, which have been shown to be detrimental to the patients’ quality of life [49,50].

Dermatologic adverse events have been shown to cause notable treatment termination and dose reduction, which might hinder clinical resolution and lead to disease progression [45]. Late dermatology consultations, if acquired, attempt to alleviate clinical symptoms of severe or persistent dermatologic adverse events; however, they may not reverse the negative connotations that patients have toward the treatment regimens, which may result in poor compliance. Moreover, late dermatology consultations will only allow melanoma treatment continuation when the dermatologic adverse events are controlled and will have a limited role in certain dermatologic adverse events that persist even after treatment termination. Therefore, a more proactive role is needed from dermatologists to screen for and manage early dermatologic adverse events to ensure maximal clinical benefit of melanoma treatment.

Furthermore, immunotherapy landmark trials excluded patients with autoimmune diseases, including autoimmune dermatitis; hence, no recommendations can be made regarding patients with ongoing autoimmune skin diseases [5,12]. However, observational studies have not only shown an exacerbation of autoimmune dermatitis, such as psoriasis, in patients undergoing immunotherapy treatment but have also reported new cases in previously healthy patients [51,52]. This alludes to the importance of integrated dermato-oncology evaluation before treatment commencement, especially in patients with ongoing autoimmune dermatitis or those predisposed to develop such diseases.

The American Academy of Dermatology recommends a collaborative approach between dermatologists and oncologists to limit treatment interruptions and improve patients’ quality of life. The academy also recommends routine dermatologic assessments to be carried out depending on the agent used, age of the patient, and predisposition to skin cancer, including any previous history of skin cancer or sun damage. In the recent American Academy of Dermatology guidelines for treating melanoma, dermatologic assessments were specifically recommended for 3 patient subgroups. First, patients on BRAFi monotherapy (targeted therapy) should be assessed every 2-4 weeks for the first 3 months. Second, patients on immunotherapy should be assessed during the first month of treatment, with additional assessments as needed. Finally, patients with autoimmune dermatitis, such as atopic dermatitis, should be assessed before therapy commencement for counseling and treatment [32]. Moreover, the US National Comprehensive Cancer Network recommends regular dermatology assessments and referrals for patients with melanoma on targeted therapy [7]. In the United Kingdom, dermatologic adverse events are managed primarily by oncologists, and there are no recommendations for routine dermatologic evaluations.

## Closing the Gap Between Dermatologists and Oncologists

Collaborative efforts between dermatologists and oncologists should be established throughout the treatment period. This is especially true because of the rapid pace of developments in advanced melanoma management, including the approval of novel agents, approval of new combinations of existing agents, and adaptation of unresectable melanoma treatments in adjuvant and neoadjuvant settings. In addition, many of these agents are widely used in other oncology disciplines. These factors contribute to the increasing patient pool, which might benefit from a more unanimous treatment approach.

Several clinical models have been implemented to improve the quality of care provided to patients with cancer, presenting with dermatologic adverse events. For instance, in North America, cutaneous oncology clinics have been established, which are run by trained dermatologists who manage dermatologic adverse events. Furthermore, several European countries have adopted dermato-oncology training programs that equip dermatologists with the means to diagnose and treat dermatologic adverse events associated with different cancer treatments. In contrast, the United Kingdom offers dermato-oncology services, such as transplant skin clinics that provide routine skin assessments that screen and manage dermatologic adverse events. However, these clinics are limited to certain tertiary hospitals, with no routine dermatology input provided in other hospitals [53].

**Figure 1:**
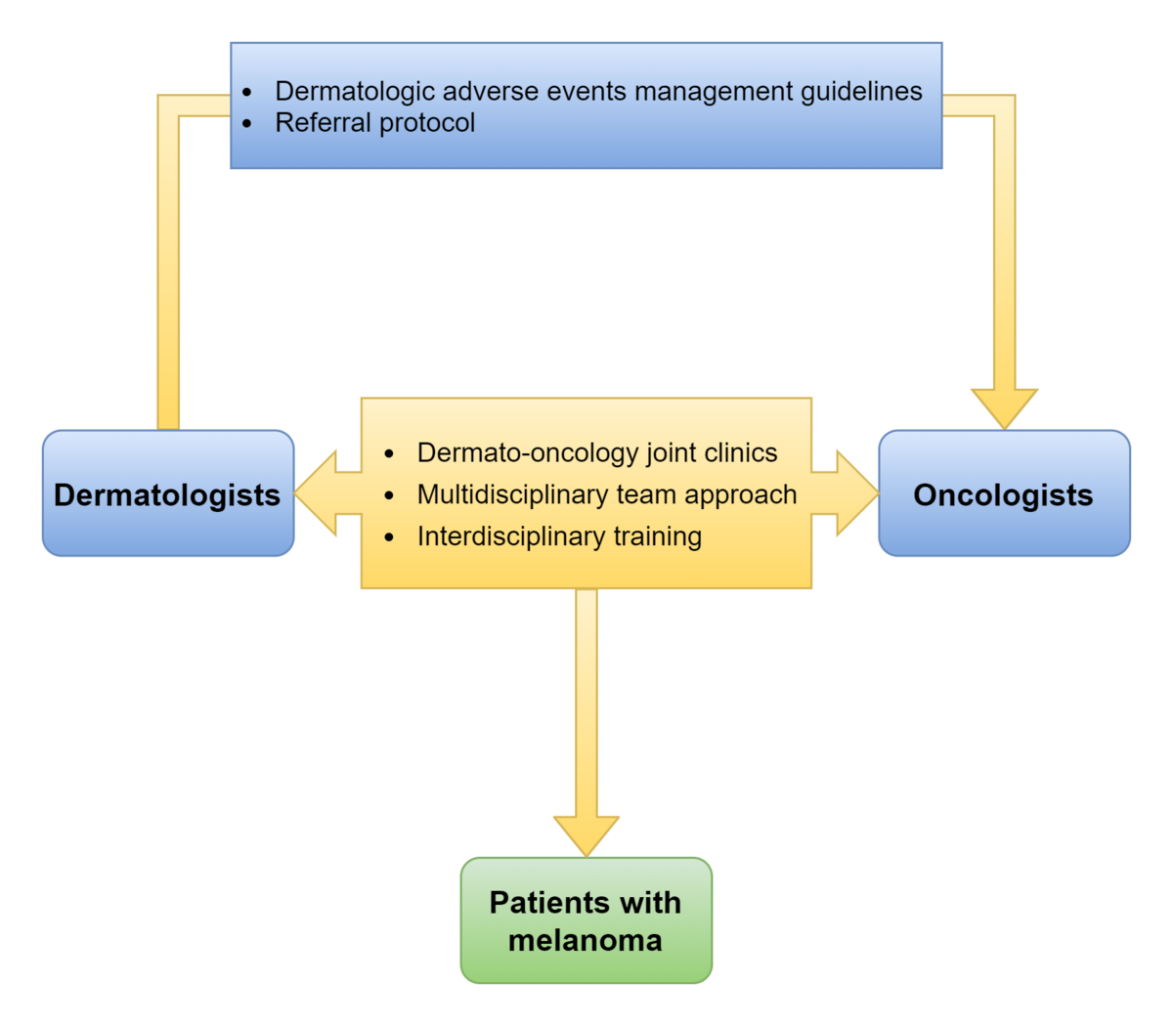
Proposed steps to improve the quality of care provided to patients with melanoma.

Several proposed steps at the institutional level, if applied, should contribute to improved and holistic care for patients with advanced melanoma (Figure 1). First, a wider range of hospitals should implement dermato-oncology joint clinics. Second, a multidisciplinary team approach should be incorporated throughout the treatment period. In addition, pretreatment dermatologic evaluations should be incorporated into the care of patients with advanced melanoma who have ongoing autoimmune dermatitis and those who are predisposed to develop such diseases. Third, dermato-oncology interdisciplinary training should be established as part of specialist training or as an independent fellowship program, which will allow the transfer of expertise between the 2 specialties. These efforts will provide dermatologists and oncologists with a better understanding of the characteristics of these agents, enabling them to recognize and manage early signs of serious dermatologic adverse events, thereby limiting unnecessary treatment interruptions.

On the departmental scale, dermatologists should formulate easy-to-follow management guidelines for common dermatologic adverse events, thus creating a higher degree of independence among oncologists when faced with dermatologic adverse events. Moreover, these guidelines should highlight the scenarios that mandate dermatology referrals, thereby facilitating the universality of care across hospitals.

## Conclusions

Because of the novelty of targeted therapy and immunotherapy, there are no mature data from head-to-head trials that compare targeted therapy and immunotherapy or delineate the role of combined or sequential targeted and immunotherapy regimens. Indirect data analyses suggest that combined targeted therapy has an advantageous therapeutic effect on rapidly developing, prognostically poor melanomas, whereas immunotherapy agents show a more durable long-term melanoma growth inhibition. Further direct comparative studies will undoubtedly offer a better understanding of the ideal treatment approach for advanced cutaneous melanoma.

Incidentally, dermatologic adverse events are among the most frequently reported adverse events with targeted therapy and immunotherapy. Because of the unclear role of dermatologists in managing dermatologic adverse events in the current guidelines, managing oncologists are faced with a plethora of treatment-related dermatologic adverse events that have been shown to be detrimental to treatment continuity and patients’ quality of life. Hence, evidence-based guidelines that incorporate dermato-oncology management are much needed to improve the quality of care provided to patients with advanced melanoma.

## Abbreviations

BRAFi: BRAF inhibitor
CTLA4: cytotoxic T-lymphocyte-associated protein 4
ERK: extracellular signal-regulated kinase
MEKi: mitogen-activated protein kinase kinase inhibitor
OS: overall survival
PD-1: programmed death 1
PD-L1: programmed death 1 ligand
PFS: progression-free survival
RCT: randomized controlled trial

## References

[1] Melanoma skin cancer statistics. Cancer Research UK.

[2] (2020). Key statistics for melanoma skin cancer. American Cancer Society.

[3] Rodríguez-Cerdeira Carmen, Carnero Gregorio Miguel, López-Barcenas Adriana, Sánchez-Blanco Elena, Sánchez-Blanco Beatriz, Fabbrocini G, Bardhi B, Sinani A, Guzman RA (2017). Advances in Immunotherapy for Melanoma: A Comprehensive Review. Mediators Inflamm.

[4] Barth A, Wanek LA, Morton DL (1995). Prognostic factors in 1,521 melanoma patients with distant metastases. J Am Coll Surg.

[5] Larkin J, Chiarion-Sileni V, Gonzalez R, Grob J, Rutkowski P, Lao CD, Cowey CL, Schadendorf D, Wagstaff J, Dummer R, Ferrucci PF, Smylie M, Hogg D, Hill A, Márquez-Rodas I, Haanen J, Guidoboni M, Maio M, Schöffski P, Carlino MS, Lebbé C, McArthur G, Ascierto PA, Daniels GA, Long GV, Bastholt L, Rizzo JI, Balogh A, Moshyk A, Hodi FS, Wolchok JD (2019). Five-year survival with combined nivolumab and ipilimumab in advanced melanoma. N Engl J Med.

[6] Michielin O, van Akkooi AC, Ascierto P, Dummer R, Keilholz U, ESMO Guidelines Committee. Electronic address: clinicalguidelines@esmo.org (2019). Cutaneous melanoma: ESMO Clinical Practice Guidelines for diagnosis, treatment and follow-up†. Ann Oncol.

[7] Swetter S, Thompson J, Coit D (2020). Cutaneous melanoma - NCCN guidelines version 3. National Comprehensive Cancer Network.

[8] Seth R, Messersmith H, Kaur V, Kirkwood JM, Kudchadkar R, McQuade JL, Provenzano A, Swami U, Weber J, Alluri KC, Agarwala S, Ascierto PA, Atkins MB, Davis N, Ernstoff MS, Faries MB, Gold JS, Guild S, Gyorki DE, Khushalani NI, Meyers MO, Robert C, Santinami M, Sehdev A, Sondak VK, Spurrier G, Tsai KK, van Akkooi A, Funchain P (2020). Systemic therapy for melanoma: ASCO Guideline. J Clin Oncol.

[9] (2020). FDA approves atezolizumab for BRAF V600 unresectable or metastatic melanoma. US Food & Drug Administration.

[10] Davies H, Bignell GR, Cox C, Stephens P, Edkins S, Clegg S, Teague J, Woffendin H, Garnett MJ, Bottomley W, Davis N, Dicks E, Ewing R, Floyd Y, Gray K, Hall S, Hawes R, Hughes J, Kosmidou V, Menzies A, Mould C, Parker A, Stevens C, Watt S, Hooper S, Wilson R, Jayatilake H, Gusterson BA, Cooper C, Shipley J, Hargrave D, Pritchard-Jones K, Maitland N, Chenevix-Trench G, Riggins GJ, Bigner DD, Palmieri G, Cossu A, Flanagan A, Nicholson A, Ho JW, Leung SY, Yuen ST, Weber BL, Seigler HF, Darrow TL, Paterson H, Marais R, Marshall CJ, Wooster R, Stratton MR, Futreal PA (2002). Mutations of the BRAF gene in human cancer. Nature.

[11] Robert C, Ribas A, Schachter J, Arance A, Grob J, Mortier L, Daud A, Carlino MS, McNeil CM, Lotem M, Larkin JM, Lorigan P, Neyns B, Blank CU, Petrella TM, Hamid O, Su S, Krepler C, Ibrahim N, Long GV (2019). Pembrolizumab versus ipilimumab in advanced melanoma (KEYNOTE-006): post-hoc 5-year results from an open-label, multicentre, randomised, controlled, phase 3 study. Lancet Oncol.

[12] Schachter J, Ribas A, Long G, Arance A, Grob J, Mortier L, Daud A, Carlino M, McNeil C, Lotem M, Larkin J, Lorigan P, Neyns B, Blank C, Petrella T, Hamid O, Zhou H, Ebbinghaus S, Ibrahim N, Robert C (2017). Pembrolizumab versus ipilimumab for advanced melanoma: final overall survival results of a multicentre, randomised, open-label phase 3 study (KEYNOTE-006). Lancet.

[13] Ascierto PA, McArthur GA, Dréno B, Atkinson V, Liszkay G, Di Giacomo AM, Mandalà M, Demidov L, Stroyakovskiy D, Thomas L, de la Cruz-Merino L, Dutriaux C, Garbe C, Yan Y, Wongchenko M, Chang I, Hsu JJ, Koralek DO, Rooney I, Ribas A, Larkin J (2016). Cobimetinib combined with vemurafenib in advanced BRAFV600-mutant melanoma (coBRIM): updated efficacy results from a randomised, double-blind, phase 3 trial. Lancet Oncol.

[14] Long G, Stroyakovskiy D, Gogas H, Levchenko E, de Braud F, Larkin J, Garbe C, Jouary T, Hauschild A, Grob J, Chiarion-Sileni V, Lebbe C, Mandalà M, Millward M, Arance A, Bondarenko I, Haanen J, Hansson J, Utikal J, Ferraresi V, Kovalenko N, Mohr P, Probachai V, Schadendorf D, Nathan P, Robert C, Ribas A, DeMarini D, Irani J, Swann S, Legos J, Jin F, Mookerjee B, Flaherty K (2015). Dabrafenib and trametinib versus dabrafenib and placebo for Val600 BRAF-mutant melanoma: a multicentre, double-blind, phase 3 randomised controlled trial. Lancet.

[15] Ascierto P, Dummer R, Gogas H, Flaherty K, Arance A, Mandala M, Liszkay G, Garbe C, Schadendorf D, Krajsova I, Gutzmer R, de Groot JW, Loquai C, Gollerkeri A, Pickard MD, Robert C (2020). Update on tolerability and overall survival in COLUMBUS: landmark analysis of a randomised phase 3 trial of encorafenib plus binimetinib vs vemurafenib or encorafenib in patients with BRAF V600-mutant melanoma. Eur J Cancer.

[16] Villanueva J, Vultur A, Lee JT, Somasundaram R, Fukunaga-Kalabis M, Cipolla AK, Wubbenhorst B, Xu X, Gimotty PA, Kee D, Santiago-Walker AE, Letrero R, D'Andrea K, Pushparajan A, Hayden JE, Brown KD, Laquerre S, McArthur GA, Sosman JA, Nathanson KL, Herlyn M (2010). Acquired resistance to BRAF inhibitors mediated by a RAF kinase switch in melanoma can be overcome by cotargeting MEK and IGF-1R/PI3K. Cancer Cell.

[17] Kakadia S, Yarlagadda N, Awad R, Kundranda M, Niu J, Naraev B, Mina L, Dragovich T, Gimbel M, Mahmoud F (2018). Mechanisms of resistance to BRAF and MEK inhibitors and clinical update of US Food and Drug Administration-approved targeted therapy in advanced melanoma. Onco Targets Ther.

[18] Griffin M, Scotto D, Josephs DH, Mele S, Crescioli S, Bax HJ, Pellizzari G, Wynne MD, Nakamura M, Hoffmann RM, Ilieva KM, Cheung A, Spicer JF, Papa S, Lacy KE, Karagiannis SN (2017). BRAF inhibitors: resistance and the promise of combination treatments for melanoma. Oncotarget.

[19] Hatzivassiliou G, Song K, Yen I, Brandhuber BJ, Anderson DJ, Alvarado R, Ludlam MJ, Stokoe D, Gloor SL, Vigers G, Morales T, Aliagas I, Liu B, Sideris S, Hoeflich KP, Jaiswal BS, Seshagiri S, Koeppen H, Belvin M, Friedman LS, Malek S (2010). RAF inhibitors prime wild-type RAF to activate the MAPK pathway and enhance growth. Nature.

[20] Gogas H, Flaherty K, Dummer R, Ascierto P, Arance A, Mandala M, Liszkay G, Garbe C, Schadendorf D, Krajsova I, Gutzmer R, Sileni VC, Dutriaux C, de Groot JW, Yamazaki N, Loquai C, Gollerkeri A, Pickard MD, Robert C (2019). Adverse events associated with encorafenib plus binimetinib in the COLUMBUS study: incidence, course and management. Eur J Cancer.

[21] Galván-Banqueri M, Ubago-Pérez R, Molina-López T (2016). The relative clinical efficacy of trametinib-dabrafenib and cobimetinib-vemurafenib in advanced melanoma: an indirect comparison. J Clin Pharm Ther.

[22] Garzón-Orjuela N, Prieto-Pinto L, Lasalvia P, Herrera D, Castrillón J, González-Bravo D, Castañeda-Cardona C, Rosselli D (2020). Efficacy and safety of dabrafenib-trametinib in the treatment of unresectable advanced/metastatic melanoma with BRAF-V600 mutation: a systematic review and network meta-analysis. Dermatol Ther.

[23] Robert C, Grob JJ, Stroyakovskiy D, Karaszewska B, Hauschild A, Levchenko E, Sileni VC, Schachter J, Garbe C, Bondarenko I, Gogas H, Mandalá M, Haanen JB, Lebbé C, Mackiewicz A, Rutkowski P, Nathan PD, Ribas A, Davies MA, Flaherty KT, Burgess P, Tan M, Gasal E, Voi M, Schadendorf D, Long GV (2019). Five-year outcomes with dabrafenib plus trametinib in metastatic melanoma. N Engl J Med.

[24] Houten R, Greenhalgh J, Mahon J, Nevitt S, Beale S, Boland A, Lambe T, Dundar Y, Kotas E, McEntee J (2021). Encorafenib with binimetinib for the treatment of patients with BRAF V600 mutation-positive unresectable or metastatic melanoma: an evidence review group perspective of a NICE single technology appraisal. Pharmacoecon Open.

[25] Dummer R, Ascierto P, Gogas H, Arance A, Mandala M, Liszkay G, Garbe C, Schadendorf D, Krajsova I, Gutzmer R, Chiarion Sileni V, Dutriaux C, de Groot J, Yamazaki N, Loquai C, Moutouh-de Parseval L, Pickard M, Sandor V, Robert C, Flaherty K (2018). Overall survival in patients with BRAF-mutant melanoma receiving encorafenib plus binimetinib versus vemurafenib or encorafenib (COLUMBUS): a multicentre, open-label, randomised, phase 3 trial. Lancet Oncol.

[26] Robert C, Karaszewska B, Schachter J, Rutkowski P, Mackiewicz A, Stroiakovski D, Lichinitser M, Dummer R, Grange F, Mortier L, Chiarion-Sileni V, Drucis K, Krajsova I, Hauschild A, Lorigan P, Wolter P, Long GV, Flaherty K, Nathan P, Ribas A, Martin A, Sun P, Crist W, Legos J, Rubin SD, Little SM, Schadendorf D (2015). Improved overall survival in melanoma with combined dabrafenib and trametinib. N Engl J Med.

[27] Long GV, Stroyakovskiy D, Gogas H, Levchenko E, de Braud F, Larkin J, Garbe C, Jouary T, Hauschild A, Grob JJ, Sileni VC, Lebbe C, Mandalà M, Millward M, Arance A, Bondarenko I, Haanen JB, Hansson J, Utikal J, Ferraresi V, Kovalenko N, Mohr P, Probachai V, Schadendorf D, Nathan P, Robert C, Ribas A, DeMarini DJ, Irani JG, Casey M, Ouellet D, Martin A, Le N, Patel K, Flaherty K (2014). Combined BRAF and MEK Inhibition versus BRAF Inhibition alone in melanoma. N Engl J Med.

[28] Delord J, Robert C, Nyakas M, McArthur GA, Kudchakar R, Mahipal A, Yamada Y, Sullivan R, Arance A, Kefford RF, Carlino MS, Hidalgo M, Gomez-Roca C, Michel D, Seroutou A, Aslanis V, Caponigro G, Stuart DD, Moutouh-de Parseval L, Demuth T, Dummer R (2017). Phase I dose-escalation and -expansion study of the BRAF Inhibitor Encorafenib (LGX818) in metastatic -mutant melanoma. Clin Cancer Res.

[29] Adelmann CH, Ching G, Du L, Saporito RC, Bansal V, Pence LJ, Liang R, Lee W, Tsai KY (2016). Comparative profiles of BRAF inhibitors: the paradox index as a predictor of clinical toxicity. Oncotarget.

[30] Koelblinger P, Thuerigen O, Dummer R (2018). Development of encorafenib for BRAF-mutated advanced melanoma. Curr Opin Oncol.

[31] Anforth R, Liu M, Nguyen B, Uribe P, Kefford R, Clements A, Long GV, Fernandez-Peñas P (2014). Acneiform eruptions: a common cutaneous toxicity of the MEK inhibitor trametinib. Australas J Dermatol.

[32] Swetter S, Tsao H, Bichakjian C, Curiel-Lewandrowski C, Elder D, Gershenwald J, Guild V, Grant-Kels JM, Halpern AC, Johnson TM, Sober AJ, Thompson JA, Wisco OJ, Wyatt S, Hu S, Lamina T (2019). Guidelines of care for the management of primary cutaneous melanoma. J Am Acad Dermatol.

[33] Ugurel S, Röhmel J, Ascierto P, Flaherty K, Grob J, Hauschild A, Larkin J, Long GV, Lorigan P, McArthur GA, Ribas A, Robert C, Schadendorf D, Garbe C (2017). Survival of patients with advanced metastatic melanoma: the impact of novel therapies-update 2017. Eur J Cancer.

[34] Kim TK, Herbst RS, Chen L (2018). Defining and Understanding Adaptive Resistance in Cancer Immunotherapy. Trends Immunol.

[35] Long GV, Menzies AM, Nagrial AM, Haydu LE, Hamilton AL, Mann GJ, Hughes TM, Thompson JF, Scolyer RA, Kefford RF (2011). Prognostic and clinicopathologic associations of oncogenic in metastatic melanoma. J Clin Oncol.

[36] Czarnecka AM, Teterycz P, Mariuk-Jarema A, Lugowska I, Rogala P, Dudzisz-Sledz M, Switaj T, Rutkowski P (2019). Treatment Sequencing and Clinical Outcomes in BRAF-Positive and BRAF-Negative Unresectable and Metastatic Melanoma Patients Treated with New Systemic Therapies in Routine Practice. Target Oncol.

[37] Patel R, Bock M, Polotti CF, Elsamra S (2017). Pharmacokinetic drug evaluation of atezolizumab for the treatment of locally advanced or metastatic urothelial carcinoma. Expert Opin Drug Metab Toxicol.

[38] Reddy SM, Carroll E, Nanda R (2020). Atezolizumab for the treatment of breast cancer. Expert Rev Anticancer Ther.

[39] Hamid O, Molinero L, Bolen CR, Sosman JA, Muñoz-Couselo E, Kluger HM, McDermott DF, Powderly JD, Sarkar I, Ballinger M, Fassò M, O'Hear C, Chen DS, Hegde PS, Hodi FS (2019). Safety, clinical activity, and biological correlates of response in patients with metastatic melanoma: results from a phase I trial of atezolizumab. Clin Cancer Res.

[40] Gutzmer R, Stroyakovskiy D, Gogas H, Robert C, Lewis K, Protsenko S, Pereira R, Eigentler T, Rutkowski P, Demidov L, Manikhas G, Yan Y, Huang K, Uyei A, McNally V, McArthur G, Ascierto P (2020). Atezolizumab, vemurafenib, and cobimetinib as first-line treatment for unresectable advanced BRAFV600 mutation-positive melanoma (IMspire150): primary analysis of the randomised, double-blind, placebo-controlled, phase 3 trial. Lancet.

[41] Ascierto PA, Ferrucci PF, Fisher R, Del Vecchio M, Atkinson V, Schmidt H, Schachter J, Queirolo P, Long GV, Di Giacomo AM, Svane IM, Lotem M, Bar-Sela G, Couture F, Mookerjee B, Ghori R, Ibrahim N, Moreno BH, Ribas A (2019). Dabrafenib, trametinib and pembrolizumab or placebo in BRAF-mutant melanoma. Nat Med.

[42] Swetter S, Thompson J (2020). Cutaneous melanoma - NCCN guidelines version 4. National Comprehensive Cancer Network.

[43] Tattersall I, Leventhal J (2020). Cutaneous toxicities of immune checkpoint inhibitors: the role of the dermatologist. Yale J Biol Med.

[44] Plachouri K, Vryzaki E, Georgiou S (2019). Cutaneous adverse events of immune checkpoint inhibitors: a summarized overview. Curr Drug Saf.

[45] Dréno B, Ribas A, Larkin J, Ascierto P, Hauschild A, Thomas L, Grob J, Koralek D, Rooney I, Hsu J, McKenna E, McArthur G (2017). Incidence, course, and management of toxicities associated with cobimetinib in combination with vemurafenib in the coBRIM study. Ann Oncol.

[46] Peuvrel L, Bachmeyer C, Reguiai Z, Bachet J, André T, Bensadoun R, Bouché O, Ychou M, Dréno B, Regional expert groups PROCUR (2013). Survey on the management of skin toxicity associated with EGFR inhibitors amongst French physicians. J Eur Acad Dermatol Venereol.

[47] Hassel JC, Kripp M, Al-Batran S, Hofheinz R (2010). Treatment of epidermal growth factor receptor antagonist-induced skin rash: results of a survey among German oncologists. Onkologie.

[48] Boone SL, Rademaker A, Liu D, Pfeiffer C, Mauro DJ, Lacouture ME (2007). Impact and management of skin toxicity associated with anti-epidermal growth factor receptor therapy: survey results. Oncology.

[49] Joshi SS, Ortiz S, Witherspoon JN, Rademaker A, West DP, Anderson R, Rosenbaum SE, Lacouture ME (2010). Effects of epidermal growth factor receptor inhibitor-induced dermatologic toxicities on quality of life. Cancer.

[50] Rosen AC, Case EC, Dusza SW, Balagula Y, Gordon J, West DP, Lacouture ME (2013). Impact of dermatologic adverse events on quality of life in 283 cancer patients: a questionnaire study in a dermatology referral clinic. Am J Clin Dermatol.

[51] Gutzmer R, Koop A, Meier F, Hassel JC, Terheyden P, Zimmer L, Heinzerling L, Ugurel S, Pföhler C, Gesierich A, Livingstone E, Satzger I, Kähler KC, German Dermatooncology Group (DeCOG) (2017). Programmed cell death protein-1 (PD-1) inhibitor therapy in patients with advanced melanoma and preexisting autoimmunity or ipilimumab-triggered autoimmunity. Eur J Cancer.

[52] Ruiz-Bañobre Juan, Abdulkader I, Anido U, León Luis, López-López Rafael, García-González Jorge (2017). Development of de novo psoriasis during nivolumab therapy for metastatic renal cell carcinoma: immunohistochemical analyses and clinical outcome. APMIS.

[53] Muthiah S, Tang D, Nasr B, Verykiou S (2018). A new era in holistic care: bridging the gap between dermatologists and oncologists for the treatment of malignant melanoma. Br J Dermatol.

